# Conscious vision in blindness: A new perceptual phenomenon implemented on the “wrong” side of the brain

**DOI:** 10.1002/pchj.787

**Published:** 2024-07-17

**Authors:** Yan Bao, Bin Zhou, Xinchi Yu, Lihua Mao, Evgeny Gutyrchik, Marco Paolini, Nikos Logothetis, Ernst Pöppel

**Affiliations:** ^1^ School of Psychological and Cognitive Sciences Peking University Beijing China; ^2^ Beijing Key Laboratory of Behavior and Mental Health Peking University Beijing China; ^3^ State Key Laboratory of Brain and Cognitive Science, Institute of Psychology Chinese Academy of Sciences Beijing China; ^4^ Department of Psychology University of Chinese Academy of Sciences Beijing China; ^5^ Program in Neuroscience and Cognitive Science University of Maryland College Park Maryland USA; ^6^ Department of Linguistics University of Maryland College Park Maryland USA; ^7^ Institute of Medical Psychology Ludwig Maximilian University Munich Munich Germany; ^8^ Department of Radiology University Hospital, Ludwig Maximilian University Munich Munich Germany; ^9^ International Center for Primate Brain Research Chinese Academy of Sciences Shanghai China

**Keywords:** blindsight, fMRI, residual vision, visual completion, visual cortex

## Abstract

Patients with lesions in the visual cortex are blind in corresponding regions of the visual field, but they still may process visual information, a phenomenon referred to as residual vision or “blindsight”. Here we report behavioral and fMRI observations with a patient who reports conscious vision across an extended area of blindness for moving, but not for stationary stimuli. This completion effect is shown to be of perceptual and not of conceptual origin, most likely mediated by spared representations of the visual field in the striate cortex. The neural output to extra‐striate areas from regions of the deafferented striate cortex is apparently still intact; this is, for instance, indicated by preserved size constancy of visually completed stimuli. Neural responses as measured with fMRI reveal an activation only for moving stimuli, but importantly on the ipsilateral side of the brain. In a conceptual model this shift of activation to the “wrong” hemisphere is explained on the basis of an imbalance of excitatory and inhibitory interactions within and between the striate cortices due to the brain injury. The observed neuroplasticity indicated by this shift together with the behavioral observations provide important new insights into the functional architecture of the human visual system and provide new insight into the concept of consciousness.

## INTRODUCTION

To get a better understanding of consciousness is a long‐term issue in psychology (e.g., Boring, 1933). With the discovery of residual vision or “blindsight” (Perenin & Jeannerod, 1975; Pöppel et al., 1973; Sanders et al., 1974; Stoerig et al., 1985; Weiskrantz et al., 1974), the reasoning about consciousness or “states of being conscious” (Pöppel, 1997a; Pöppel, 1997b) has added a new level to discussions about consciousness (e.g., Block, 1995; Dennett, 1991; Weiskrantz, 1997). An important new aspect is that the phenomenon of blindsight emphasizes the importance of implicit knowledge (Bao et al., 2022) and how this relates to an expanded concept of consciousness. Such a broader view follows also from a taxonomy of psychological functions in which a distinction is made between content and logistic functions (Zhao et al., 2022): content functions (like percepts or memories) have a conscious quality whereas logistic functions (like temporal control) are lacking this quality. However, temporal control as a pre‐semantic logistic function is crucial for states of being conscious and temporal continuity of conscious activities (Izadifar, 2021; Pöppel, 1997a; Pöppel, 1997b; Pöppel & Bao, 2014). The importance of the visual cortex for conscious vision has been stressed before (Zhou et al., 2016), i.e. neural processing in this primary visual processing area is essential to conscious vision at all. In a unique single case study with a brain‐injured patient, which has never been done before, this claim has obtained substantial support.

Residual vision or “blindsight” as a perceptual capacity after an acquired brain injury has been proven to be a valid phenomenon (e.g., Cowey, 2010; Kinoshita et al., 2019). After suffering a lesion in the visual cortex or in the projection pathway to the visual cortex, patients appear to be absolutely blind in corresponding areas of the visual field on the conscious level, but they can still detect and also discriminate visual stimuli without conscious representation. Some early criticism that this phenomenon might be explained by scattered light (Campion et al., 1983) can be rejected (Pöppel et al., 1973; Zihl & Werth, 1984).

**FIGURE 1:**
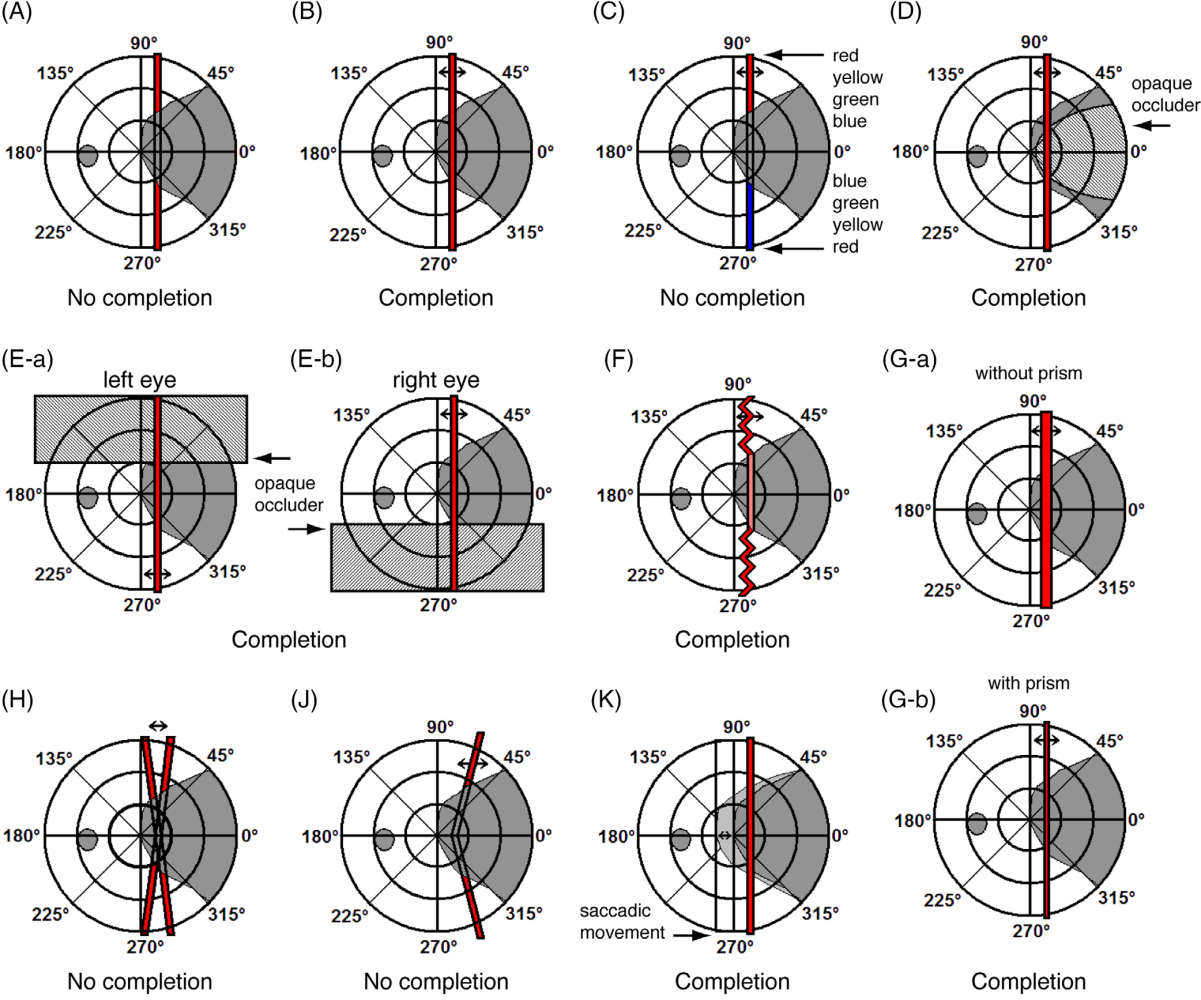
The visual field of patient FS with a blind area (dark grey, wedge‐shaped) shows perceptual completion or lack of completion under different experimental conditions. (A) Stationary vertical stimulus across the blind area; *no completion*. (B) Moving stimulus parallel to the vertical meridian; *completion*. (C) Moving stimulus with different colors above and below the blind area; *no completion*. (D) Moving stimulus behind an opaque occluder, i.e. no physical stimulus within the blind area was present; *completion*. (E‐a and E‐b) Moving visual stimulus for the left eye in the right lower, for the right eye in the right upper quadrant; *completion*. (F) Zigzag pattern of a moving stimulus; *vectorial completion*. (G‐a and G‐b) Moving visual stimulus with different states of visual vergence and accommodation; *completion with different apparent size of the stimulus*. (H) Pendular movements of the stimulus; *no completion*. (J) Moving stimulus not parallel to the vertical meridian and with local information within the scotoma; *no completion*. (K) Induction of stimulus motion across the retina by lateral saccadic eye movements; *completion*.

One of the most intensely studied patients demonstrating residual vision or blindsight is FS (Cowey, 2010; Stoerig et al., 1998; Stoerig & Cowey, 1997), a male patient, who has a blind area (scotoma) in the right visual field with remaining visual perception above and below the scotoma (Figure 1). The areas affected by the brain injury have been well documented (Stoerig et al., 1998). Patient FS, born in 1937, had an accident in 1979, and he suffered a severe cranio‐cerebral trauma in the left hemisphere resulting in an incomplete hemianopia in the right visual field; the right hemisphere on a macroscopic level was unaffected by the accident. Patient FS displays an unexpected phenomenon of conscious vision within the scotoma, i.e., he reports seeing a continuous trajectory of a moving visual stimulus across an extended blind region of the visual field. This phenomenon of visual completion reported in a nutshell already some time ago (Pöppel, 1985) provided a unique opportunity to study over the years structural and functional mechanisms of the human visual system on the behavioral level and finally also with imaging technology.

## MATERIALS AND METHODS

Perimetric visual field measurements were repeatedly performed with patient FS under monocular and binocular viewing conditions for some 30 years. They served as a basis for the behavioral observations and fMRI measurements. The visual field mapping was done with a Tübinger perimeter (Aulhorn & Harms, 1972). The patient fixated a continuously presented red spot in the center of the sphere with a diameter of 30 min arc and a luminance of 32 cd/m^2^. The perimetric visual field measurement was done under photopic adaptation conditions with a homogeneous background luminance of 3.2 cd/m^2^. For the mapping of the visual field, a white stimulus is used with a luminance of 320 cd/m^2^. The test stimulus was moved with a velocity of approximately 2 deg/s along different meridians of the visual field to determine the border between blindness and seeing. The measurements were done both under binocular and monocular viewing conditions for each eye independently, in which case the unexamined eye was covered with an opaque occluder. A special optical construction of the perimeter allowed the control of fixation by the experimenter. The perimetric measurements, which were repeated many times, confirmed the long‐term stability of the area of blindness.

In the experiments of visual completion, which were repeated many times, the patient fixated a target, and different visual stimuli were presented in the visual field either on the side of the scotoma or also on the unaffected side. Either stationary or moving stimuli were presented, and the local features above and below the scotoma were systematically modified. For moving stimuli, the velocity was varied at different sessions between jerky fast and slow movements. The patient had to report whether he saw an uninterrupted stimulus across the scotoma (completion) or two isolated segments above and below the scotoma (no completion). In addition to reporting verbally what he consciously had perceived, he was asked to draw what he had seen. The observations obtained for more than three decades by different observers can statistically be treated as independent measurements. Using a binomial test with a null hypothesis that specific stimulus configurations show randomly completion or no completion, the probability to obtain consistent results across successive measurements (*N* = 20 under conservative assumptions) can be estimated as *p* = 2^−20^ << .001. Thus, the null hypothesis can be rejected, and our observations on the statistical level can be considered as reliable.

On the basis of the behavioral data, an experimental protocol was developed for fMRI measurements. The study was approved by the local ethics committee of the medical faculty of Ludwig Maximilian University Munich, and all experiments were performed in accordance with the guidelines of the Declaration of Helsinki. Two independent brain scans were performed showing the same results; only one data set is shown here. The data of the fMRI experiments focusing on the visual cortex were analyzed independently, and both analyses led to the same conclusions.

## RESULTS

### Behavioral observations of visual completion

The observations described below were made both under monocular and binocular viewing conditions. The panels in Figure 1 show only the results of stimulating the nasal visual field of the left eye up to an eccentricity of 30 degrees. The patient reported seeing separate parts of a vertically extended stimulus in the right upper and lower quadrants when the visual stimulus was presented across the scotoma. However, this occurred only if a *stationary* stimulus was presented (Figure 1A). When the stimulus was *moved* (Figure 1B), the patient reported seeing the entire stimulus across the scotoma straddling an area of more than 20 degrees visual angle.

If the color above and below the scotoma was different, completion was never reported (Figure 1C), i.e., a uniform color was necessary to construct a perceptual trajectory across the scotoma. This observation implies the existence of lateral connections which process the same spectral components of light. Such connections at the retinal level without orientation selectivity have been suggested previously using color induction as indicator (Pöppel, 1986).

To examine potential parallel pathways, the stimulus was moved behind an opaque occluder and, thus, was not physically present within the scotoma. Completion was also reported under such conditions (Figure 1D). Therefore, completion is probably of cortical origin associated with stimulation by the upper and lower parts of the visual field as projections for parallel pathways corresponding to the occluded part of the visual field do not receive direct optical input. Geniculate inputs to spared regions in the cortex are most likely responsible for the observed effect.

The spatial configuration of the area of blindness in the visual field enabled us to examine potential binocular fusion, i.e., it was possible to present the stimulus above the scotoma to one eye and below the scotoma to the other eye (Figure 1E‐a, E‐b). Completion was again reported. This indicates that completion is presumably implemented at the level of binocular fusion of visual input and not earlier in the visual pathway; this on the neural level is most likely beyond layer IV in the striate cortex (e.g., Hubel & Wiesel, 1968).

Interestingly, zigzag patterns above and below the scotoma result in vectorial completion (Figure 1F). The patient reported seeing a straight line instead of a pattern as presented within the functional regions of the visual field. This observation indicates that the global pattern movement is decisive and not the local orientation of some moving details of the stimulus. A contribution of higher visual‐motion areas, such as the medial temporal complex (MT/V5), is suggested because a substantial number of MT/V5 neurons are sensitive to the direction of global motion (Smith et al., 2005). However, some V1 neurons also respond to pattern motion, possibly mediated by feedback inputs from the extra‐striate cortex, such as MT/V5 (Guo et al., 2004).

Importantly, the stimulus had to be moved parallel to the vertical meridian; pendulum movements or oblique movements did not result in completion (Figure 1H). If parts of the stimulus above and below the scotoma were tilted so that they no longer were parallel to the vertical meridian, no completion was reported (Figure 1J). Thus, completion only occurs when local information outside the scotoma indicates vertical continuity.

If the upper part of the vertical stimulus is laterally displaced with respect to the lower part, and the stimulus no longer represents an almost exact physical continuity, completion is observed up to approximately 1 degree visual angle displacement, but not beyond; thus, the stimulus has to satisfy a rather precise vertical trajectory. This observation allows an indirect estimate of an upper limit of the receptive‐field size of neurons representing the visual field parallel to the vertical meridian. The implied size of receptive fields, which are presumably involved in completion, corresponds to equivalent measurements for the striate cortex in humans and nonhuman primates, but not for receptive fields beyond this area in the extra‐striate cortex like in the MT/V5 complex (Yoshor et al., 2007).

Size constancy for visual targets at short distances is based on visual vergence and accommodation (von Holst, 1955). The apparent size of a stimulus can be changed by using prisms to alter the visual axes of the two eyes to modulate convergence. The patient reported a different size of the completed stimulus with an altered angle of the visual axes (Figure 1G‐a, G‐b). The size of the stimulus at the same distance appeared as expected to be smaller with increased vergence of the visual axes. This observation suggests a functional output to extra‐striate areas of the visual brain which provide a coordinate transformation based on neural information coming from extraocular muscles implementing neuronally size constancy (Pöppel, 1988).

We also tested whether stimuli had to be physically moved in visual space, or whether it was sufficient to simply create a spatial displacement across the retina (Figure 1K). If the stimulus was kept stationary and retinal displacement was induced by lateral saccadic eye movements between two fixation points such that the stimulus remained optically within the scotoma for the two fixation conditions, completion was also obtained. This observation implies that cortical processing at this level is not derived from external space. It is a mere bottom‐up process without any top‐down control, the physical parameters being sufficient to create perceptual continuity. Thus, the completion is not of “conceptual” nature.

Visual completion was found to be limited to the perifoveal region up to an eccentricity of approximately 10–15 degrees, supporting the notion of a functional subdivision of the visual field. Such inhomogeneity has been demonstrated for the anatomical projections in non‐human primates (Wilson & Toyne, 1970), temporal processing (Zhou et al., 2010), or inhibition of return which suggests different attentional systems in the visual field (Bao et al., 2013; Bao & Pöppel, 2007; Lei et al., 2012). The limitation of visual completion to the perifoveal region also for the attentional machinery strongly supports the general concept of neuroanatomical constraints of higher‐level perceptual processes. A functional subdivision is also supported by resting‐state functional connectivity MRI, which shows a division of the early visual cortex into central and peripheral systems (Yeo et al., 2011).

Several points of our behavioral observations have to be emphasized: Completion was only observed for vertically extended stimuli when they were moved horizontally up to an eccentricity of approximately 10 degrees. This phenomenon is different from completion across the blind spot, as blind spot completion is not orientation selective; and the blind spot is also located more peripheral to the region of the visual field where we observed completion. Furthermore, the completion in our case cannot be explained as representing the phenomenon of virtual contours (“Kanisza figures”) as these contours are not orientation or movement dependent. Finally, completion in our case is not a “conceptual” but a low‐level “perceptual” phenomenon as documented, for instance, by the fact that a retinal displacement provoked by lateral saccadic eye movements also results in completion; this indicates that we are dealing in this case with a mere bottom‐up process with no top‐down control.

As a control we tried to simulate the completion phenomenon in 30 healthy adults by occluding the central part of a moving bar and mimicking the experimental situation with the patient. The stimulus parameters corresponded to those used with the patient. In no case did an observer report that the two fragmented parts were completed with perceptual quality, i.e., seeing the central part of the bar. Instead, subjects reported that they knew the bar was a continuous trajectory, but did not perceive the stimulus within the occluded part. An equivalent control was done with patient FS himself in his intact visual hemi‐field, and he reported and confirmed by drawing, that for the occluded condition in this hemi‐field the perceptual experience was different from what he saw in the hemi‐field with the scotoma, i.e., there was no completion in his functional visual half‐field.

### Neural observation of visual completion

**FIGURE 2:**
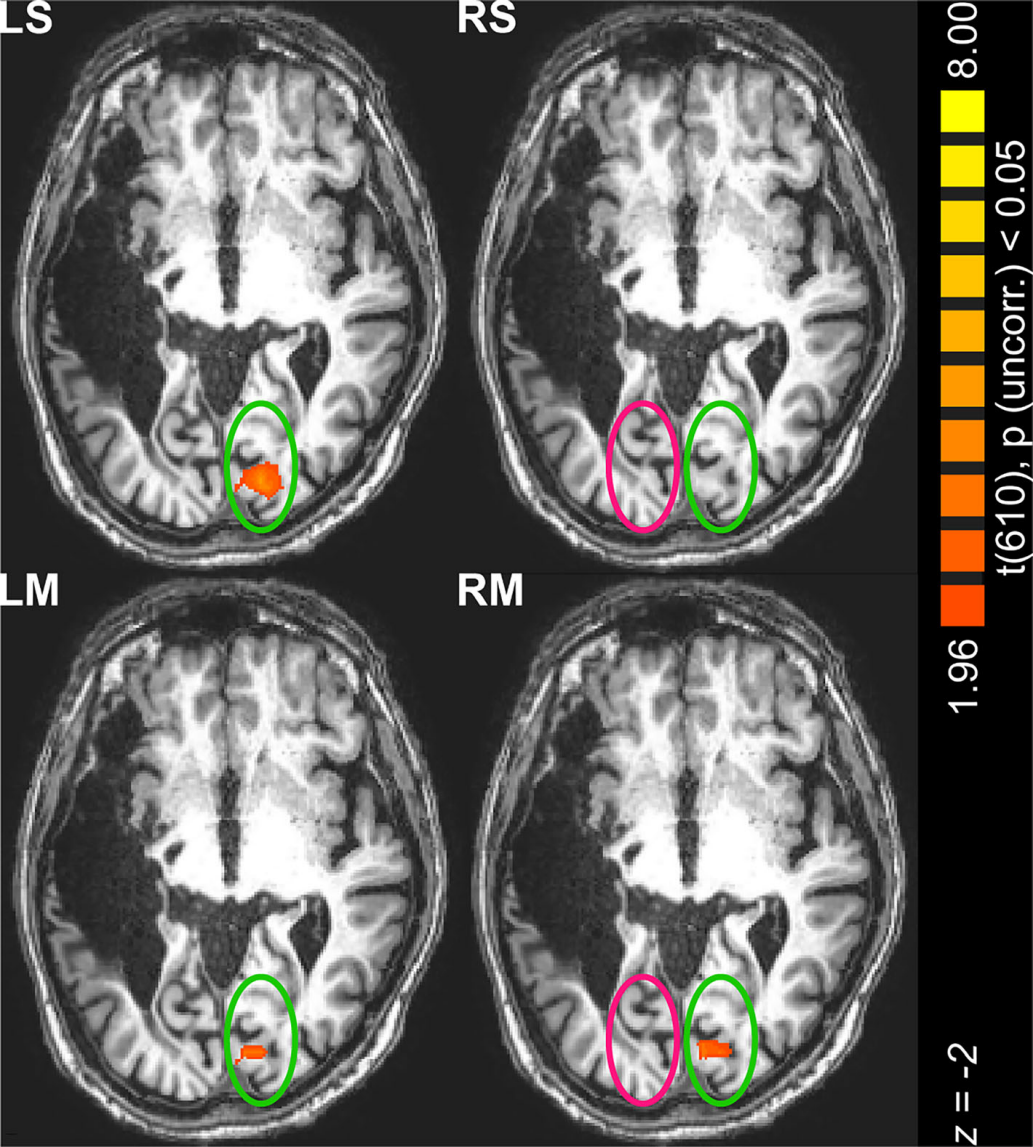
Neurofunctional correlates of visual completion in the visual cortex of patient FS measured by fMRI. Stationary (LS) and moving (LM) stimuli in the *left* visual hemi‐field activate the *right* (contralateral) visual cortex, whereas only moving (RM), but not stationary (RS), stimuli in the *right* visual hemi‐field result in activation in the *right* (ipsilateral) visual cortex. The statistical parametric map is derived from the contrast of experimental conditions > baseline and is mask‐restricted to the visual cortex. The *z* coordinate is in the Talairach stereotaxic space. Neurological convention (left is left) is used.

Is there direct evidence for an involvement of cortical structures for conscious vision, as suspected on the basis of the behavioral observations? We measured neural local activations with functional magnetic resonance imaging (fMRI) when the patient viewed a vertical bar in his left or right visual field. The bar was either stationary or moved parallel to the vertical meridian within 10 degrees eccentricity and straddled the scotoma. For such an experimental task, using fMRI is considered to be an acceptable technology (Logothetis, 2008). Results indicated that only moving, but not stationary, stimuli of identical color for upper and lower halves across the scotoma activated the visual cortex. This was observed, however, on the ipsilateral side, but not, as expected on the basis of the well‐known projection of the visual field to the striate cortex and the rigidity of this projection (Pöppel et al., 1987), on the contralateral side. An unexpected ipsilateral activation has also been observed in another case (Tran et al., 2019), however, bypassing the striate cortex (V1) which is fundamentally different from our case. Stimuli presented in the intact left visual field elicited similar BOLD signals in the contralateral hemisphere (Figure 2, Table 1, and Supporting Information) for both moving and stationary stimuli.

**TABLE 1:** Areas showing activation to bar stimuli in the occipital visual cortex.

Brain region	*x*	*y*	*z*	*t*	mm^3^
LS					
Right V1	12	−85	−5	5.54	894
Right V2	12	−83	−3	4.12	293
LM					
Right V1	12	−85	−2	3.65	228
RS					
No activation					
RM					
Right V1	6	−84	−2	3.50	192
RCM					
No activation					

*Note*: *x*, *y*, *z* coordinates are in the Talairach space (voxel with peak *t*‐score in a given region); *t*‐scores significant by *p* < .05 (uncorrected).

Abbreviations: LS, stationary stimuli in the left hemifield; LM, moving stimuli in the left hemifield; RS, stationary stimuli in the right hemifield; RM, moving stimuli in the right hemifield; RCM, moving stimuli with different colors in the right hemifield; mm^3^, cluster size.

## DISCUSSION

The activation on the ipsilateral side by moving stimuli comes as a surprise, but it may be plausible when considering a substantial divergence of projection (Rockland & Knutson, 2001) and an ipsilateral representation of the visual field due to callosal connections between the two hemispheres (Kennedy et al., 1986; Payne, 1990). In non‐human primates, an ipsilateral representation of the visual field along the vertical meridian is less than a few degrees in the perifoveal region, but reaches more than 10 degrees in the distant periphery. This mode of representation could enable fragments of the moving bar outside the scotoma to be represented also in the intact ipsilateral hemisphere.

**FIGURE 3:**
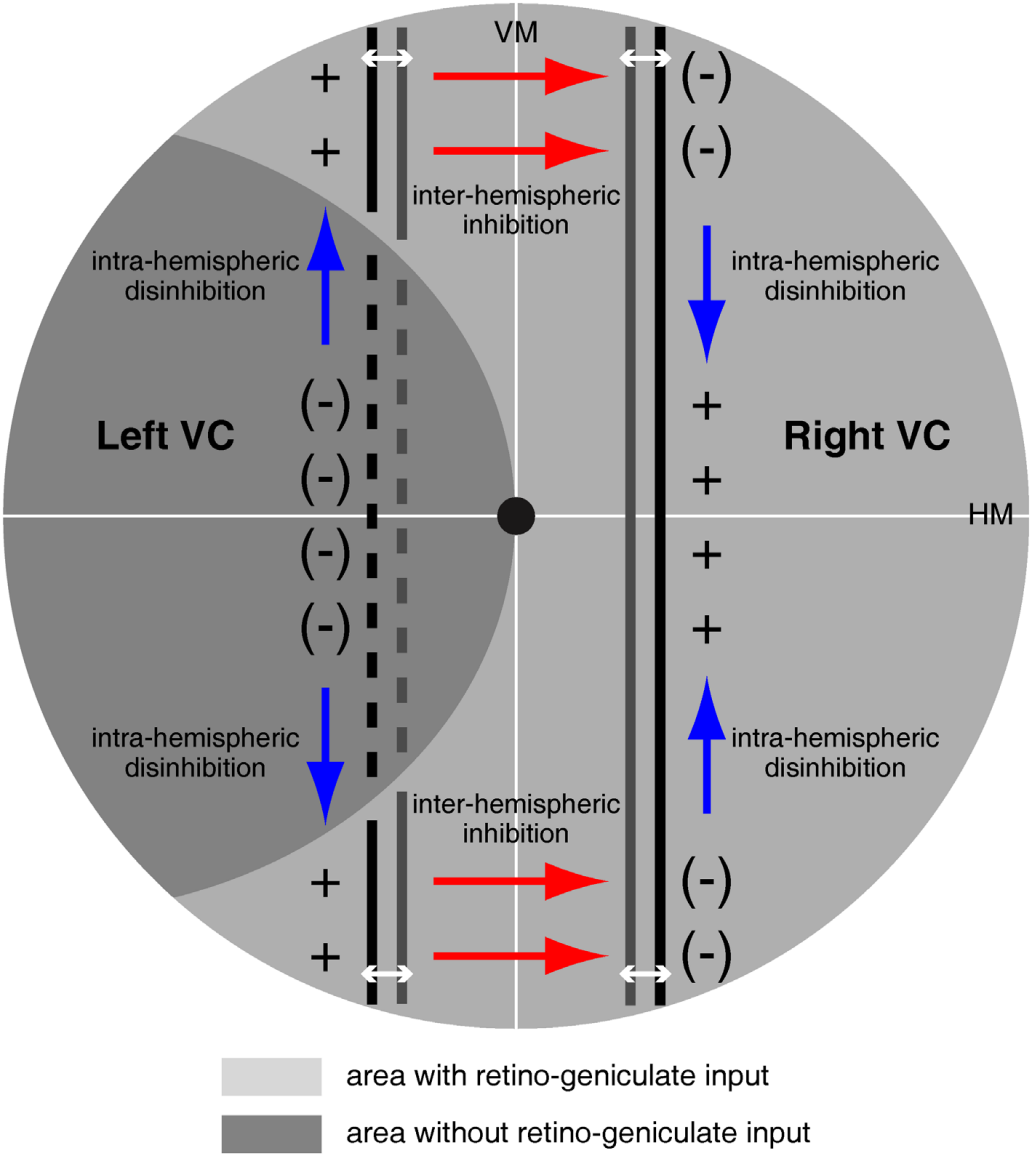
Conceptual model for the shift in neural activation from the contralateral to the ipsilateral hemisphere as indicated in Figure 2. The missing input into the left visual cortex (VC) (darker grey area; indicated by minus signs in parentheses) results in disinhibition between neighboring neural elements (indicated by the blue arrows). This reduced inhibition releases higher neural activity (indicated by plus signs) in the neighboring upper and lower parts (within the lighter grey areas). The higher neural activity inhibits neural elements across the vertical meridian (VM) at mirror‐symmetric positions in the right VC (indicated by the red arrows above and below the horizontal meridian; HM). This inhibition (indicated by minus signs in parentheses) releases higher neural activity (indicated by the plus signs and the blue arrows) filling in the missing retino‐geniculate input. The different local inhibitions and disinhibitions triggered by vertical stimuli moving horizontally are suspected to provide the neural basis for perceptual completion within a mirror‐symmetric region in the contralateral hemisphere of the visual cortex.

We suggest a conceptual model which may account for visual completion as described in this paradigmatic case (Figure 3): Retino‐geniculate input to spared regions of the visual cortex selects a distinct neural program for moving stimuli. The selection between moving and stationary targets shows a strong bias for vertical contours. Such an orientation bias has also been demonstrated in single neurons (Innocenti, 2009; Schmidt et al., 2010), and it has been observed in a brain‐injured patient who reported a vertical elongation of squares being perceived as rectangles (Dilks et al., 2007). The missing input to the left visual cortex results in an imbalance between excitatory and inhibitory interactions of neighboring units. Therefore, the neural representations of the vertical stimulus from the upper and lower visual fields on the injured side attain a higher excitatory state due to the lack of inhibition from neighboring units not receiving retino‐geniculate input. The callosal projections are assumed to be inhibitory (Bocci et al., 2011; Innocenti, 2009; Innocenti et al., 2022). As a consequence, the mirror‐symmetric population of neurons in the right ipsilateral hemisphere is inhibited, and their inhibition on neighboring units is consequently reduced; mirror‐symmetric or homologous connections have also been observed in another case (Celeghin et al., 2017). This initiates a neural “travelling wave” across the cortical surface along the privileged representation of vertical contours in the right hemisphere. Such a mechanism with respect to a directed flow of neural information has already been suggested, for instance, for binocular rivalry in the visual cortex (Lee et al., 2005). The neural filling‐in in the right ipsilateral visual cortex is further enhanced by a reduction of interhemispheric inhibition from the injured area of the left hemisphere.

Taken together, the neural mechanism for a shift in activity from the contralateral to the ipsilateral side consists of a sequence of intra‐hemispheric disinhibitions, inter‐hemispheric inhibitions, and again intra‐hemispheric disinhibitions. On a more general level, the observed neural plasticity indicated by the hemispheric shift appears to be initiated by local imbalances between excitatory and inhibitory circuits within the neural machinery. The results further indicate that neural processing on the primary visual cortex is crucial for conscious vision, and that to reach that goal, even the “wrong” hemisphere can take over.

## CONFLICT OF INTEREST STATEMENT

The authors declare no conflicts of interest.

## ETHICS STATEMENT

The study was approved by the local ethics committee of the medical faculty of Ludwig Maximilian University Munich, and all experiments were performed in accordance with the guidelines of the Declaration of Helsinki.

## Supporting information


**Data S1:** Supporting Information.
