# Supplementary material for: Conscious vision in blindness: A new perceptual phenomenon implemented on the “wrong” side of the brain
Source: Psych J. 2024 Jul 17;13(6):885–92. doi: 10.1002/pchj.787 (PMC11608789; doi:10.1002/pchj.787)
Supplement: Supplementary file 1 — Data S1: Supporting Information. [file PCHJ-13-885-s001.docx]

**Supplementary Material on fMRI measurements**

In the fMRI measurements under photopic adaptation conditions the stimulus presentation and the experimental procedure were controlled by Presentation software (Neurobehavioral Systems, Albany, NY). A trial started with a central fixation cross (duration = ~1000 ms), followed by a ~3260 ms display of a vertical bar (~0.77° × 59°) which was centered on the horizontal meridian. The bar was either stationary at the eccentricity of ~8.46° visual angle or moved horizontally from ~6.92° to ~10° away from the vertical meridian at a speed of ~10°/sec. Five conditions were measured: a stationary (LS) or a moving (LM) bar in the left (intact) visual hemifield, a stationary (RS) or moving (RM) bar in the right hemifield, and a moving bar with an upper part in red and lower part in green in the right hemifield (RCM). The measurements were conducted in five runs of 2 blocks for each condition (10 blocks in total). Each block contained four trials (resulting in 40 trials for each condition during the experiment). The presentation order of blocks was counterbalanced among runs. The patient was lying comfortably in the scanner supported by foam cushions to minimize head movement and was instructed to continuously view the screen and to fixate the central cross. The patient was encouraged to take short breaks between runs.

The data (Fig. 2) were collected on a 3-T whole-body system (Philips ACHIEVA, Germany). They were acquired by a T2*-weighted echo-planar imaging (EPI) sequence with whole-brain imaging in axial orientation (TR = 2500ms, TE = 30ms, FA = 90°, number of slices = 52, slice thickness = 3 mm, no inter-slice gap, ascending acquisition, FOV = 237 × 237 mm, matrix = 144 × 144, in-plane resolution = 1.65 × 1.65 mm). Anatomical images were obtained by a T1-weighted, magnetization-prepared rapid gradient echo (MPRAGE) sequence (TR = 8.18 ms, TE = 3.72 ms, FA = 8°, number of slices = 220, slice thickness = 1 mm, FOV = 240 × 240 mm, matrix = 256 × 256, no inter-slice gap) for anatomical reference and detection of potential morphological anomalies. Data analysis was conducted using the commercial software BrainVoyager QX 2.0.7 (BrainInnovations BV, Maastricht, The Netherlands). The T1 saturation effect was controlled by discarding the first five volumes of each run. In the pre-processing step, a six-parameter rigid-body trilinear interpolation and a high-pass filter with a cutoff of two cycles in the time course were performed to correct for three-dimensional motion and to remove low-frequency drifts, respectively. The data was realigned to the first volume, which was subsequently aligned to the anatomical images. The resultant data were normalized into the Talairach stereo-tactic space before being re-sampled to the resolution of 3 × 3 × 3 mm^3^/voxel. Statistical analysis was performed on normalized BOLD signals whose time course was z-transformed. Blocks of 17 sec corresponding to four trials of one condition were modeled with boxcar regressors and convolved with the hemodynamic response function (two gamma hemodynamic response function [HRF] with onset = 0 s; time to response peak = 5 s; time to under- shoot peak = 15 s). Five conditions, i.e. LS, LM, RS, RM, and RCM, were contrasted with respective baseline periods preceding the corresponding stimulus blocks, where only the fixation cross was presented. Statistical maps for the specific contrasts were calculated as t-statistics on a voxel-wise basis and were mask-restricted to the visual cortex to examine whether early cortical structures are involved in the perceptual completion of a moving stimulus across an acquired scotoma. Two independent brain scans were performed showing the same results; only one data set is shown here. The data of the fMRI-experiments focusing on the visual cortex were analyzed independently, and both analyses led to the same conclusions.
